# The International Congress

**Published:** 1899-02

**Authors:** 


### THE INTERNATIONAL CONGRESS.

The International Homœopathic Congress will hold its next meeting at Paris, in 1900. It was decided at the meeting in London, in 1896, to antedate the next meeting by one year, in order to make it coincide with the Exposition Universelle, in 1900.

The Société Française d'Homœopathie has accepted the task of organizing the Congress, and has appointed a commission for the purpose. It has also obtained from the Management of the Exposition, a place among the Official Congresses meeting in connection therewith.

The date of the Congress is not yet fixed, but will be sometime between July 20th and August 19th, 1900.

The co-operation of the whole profession is desired. Essays for discussion are needed, and representatives of the homœopathic system are desired to conduct these discussions. The members of the commission referred to above are P. Joussett, President; R. Hughes, Permanent Secretary; Victor Chancerel, A. Gonnard, Marc Jousset, J. Love, and J. P. Tessier.

All essays and papers should be sent before January 1st, 1900, to Dr. Léon Simon, 24 Place Vendôme, Paris, France.

